# Supplementary material for: PCR Test Positivity and Viral Loads during Three SARS-CoV-2 Viral Waves in Mumbai, India
Source: Biomedicines. 2023 Jul 8;11(7):1939. doi: 10.3390/biomedicines11071939 (PMC10377402; doi:10.3390/biomedicines11071939)
Supplement: Supplementary file 1 [file biomedicines-11-01939-s001.zip › biomedicines-2440229-supplementary.pdf]

PCR test positivity and viral loads during three SARS-CoV-2 viral waves in Mumbai, India

Supplementary web appendix

Supplementary Tables

Table S1 - Mumbai ward wise slum and non-slum population
Table S2 - Mumbai postal pin codes mapped to municipal wards
Table S3 - ICMR approved testing kits used at Thyrocare central laboratory for PCR diagnosis of SARS-CoV-2 infection
Table S4 - PCR confirmed COVID cases and deaths of Mumbai COVID dashboard at different viral
Table S5 - Descriptive PCR positivity of the Thyrocare tested population at different viral outbreak periods from April 2020 to January 2022
Table S6 - Descriptive Ct values of the Thyrocare tested population at different viral outbreak periods from April 2020 to January 2022
Table S7 - The begin, peak and end of viral waves observed in Ct values, PCR positivity, confirmed COVID case and deaths at Delta and Omicron outbreaks

Supplementary Figures

Figure S1 - Age trend of PCR positivity during Aleph, Delta, and Omicron viral waves
Figure S2 - Overview of Mumbai SARS-CoV-2 viral outbreaks - daily time series of MCGM COVID-19 dashboard and Thyrocare study data
Figure S3 - Vaccine coverage, daily time series of PCR confirmed cases and deaths from COVID-19 from January 2021 - January 2022 in Mumbai

Table S1 - Mumbai ward wise slum and non-slum population

Ward name	Population 000					
	Total	Male	Female	Slums	Non-slums	% in slums
Low slum density wards						
A	185.0	101.2	83.8	22.3	162.7	12%
B	127.3	70.4	56.8	12.7	114.6	10%
C	166.2	98.0	68.1	16.6	149.6	10%
D	346.9	183.0	163.9	34.7	312.2	10%
E	393.3	216.1	177.2	124.2	269.1	32%
G South	377.7	331.2	267.9	124.3	253.4	33%
H West	307.6	305.9	251.4	82.6	225.0	27%
K West	748.7	401.5	347.2	215.7	533.0	29%
R Central	562.2	289.2	273.0	172.8	389.3	31%
T	341.5	176.2	165.3	85.6	255.9	25%
Medium slum density wards						
F North	529.0	285.2	243.9	238.1	290.9	45%
F South	361.0	190.7	170.2	180.1	180.8	50%
G North	599.0	208.8	169.0	361.7	237.4	60%
M West	411.9	217.6	194.3	165.0	246.9	40%
N	622.9	332.6	290.2	249.2	373.6	40%
P South	463.5	249.5	214.0	230.8	232.7	50%
R South	691.2	379.3	311.9	414.4	276.8	60%
S	743.8	398.9	344.9	408.4	335.3	55%
High slum density wards						
H East	557.2	161.7	145.9	388.9	168.3	70%
K East	823.9	440.7	383.2	572.8	251.1	70%
L	902.2	500.6	401.6	758.1	144.1	84%
M East	807.7	436.6	371.2	686.0	121.7	85%
P North	941.4	507.5	433.8	708.2	233.1	75%
R North	431.4	233.7	197.7	281.2	150.2	65%
Total	12,442.4	6,716.0	5,726.4	6,534.5	5,907.9	53%

- Mumbai's municipal ward-wise population at 2011 government census. More than half of the population in Mumbai resides in slums.
- A, B, C, D, and E wards are in Mumbai city district and others in Mumbai suburban district.
- Wards with up to 33% of their population in slums are classified as low slum density areas. These are wards A, B, C, D, E, H-West, K-West, G-South, R-Central and T which together consist about 29% of the city's population. An average of 25% of the population of these wards lives in slums.
- Wards with 61% or more of their population in slums are classified as high slum density areas. These are the wards R-North, K-East, H-East, P-North, L and M-East, which consist about 36% of the city's population. An average of 76% of the population of these wards lives in slums.
- The remaining wards (N, M-West, F-South, F-Nort, S, R-South, G-North and P-South) consist of another 36% of the city's population are classified as the medium-slum density areas. 51% of the population of these wards lives in slums.

Source: https://github.com/muradbanaji/MumbaiData/blob/master/SlumNonslum.pdf

Table S2 - Mumbai postal pin codes mapped to municipal wards

Mumbai city district		Mumbai Suburban district	
Ward name	Pin code	Ward name	Pin code
A	400001	HE	400029
	400005		400051
	400020		400055
	400021		400098
	400032	HW	400050
	400039		400052
B	400003		400054
	400009	KE	400057
C	400002		400059
D	400004		400060
	400006		400069
	400007		400093
	400026		400096
	400034		400099
	400035	KW	400049
E	400008		400053
	400010		400056
	400011		400058
	400027		400102
FN	400014	L	400024
	400019		400070
	400022		400072
	400031		400084
	400037	ME	400043
FS	400012		400085
	400015		400088
	400033		400094
GN	400016	MW	400071
	400017		400074
	400028		400089
GS	400013	N	400075
	400018		400077
	400025		400079
	400030		400086
		PN	400061
			400064
			400095
			400097
		PS	400062
			400063
			400065
			400104
		RC	400091
			400092
		RN	400068
			400103
		RS	400067
			400101
		S	400042
			400076
			400078
			400083
			400087
		T	400066
			400080
			400081
			400082

Notes: Out of the 91 designated pin codes in Mumbai, some straddled more than one municipal wards. We mapped those pin codes into the municipal ward based on population.

Table S3 - ICMR approved testing kits used at Thyrocare central laboratory for PCR diagnosis of SARS-CoV-2 infection

No.	Test Kit Name	Targets (Genes)*	LOD (Limit of Detection)
1	RealStar®SARS-CoV-2PCRKit	S, E	0.014,0.025 PFU/ml
2	Lab gun Siemens	RdRp, E	20 genomic RNA copies/µL
3	TaqPath™COVID-19 Combo Kit	S, ORF1ab, N	10 GCE/reaction
4	Detection expert1SSARS CoV-2 Gene store	N1, N2	100 GCE/Reaction
5	MerilCOVID-19One-stepPCRKit	N, ORF1ab	<500 RNA copies/mL
6	COVIPATH	N, ORF1ab	10 GCE/reaction

Source: Thyrocare laboratory.
Abbreviations: PFU, plaque forming units; GCE, Genomic copy equivalents; mL, milliliter; RNA, Ribonucleic acid; µL, microliter.
*Details on gene targets can be found at https://www.who.int/docs/default-source/coronaviruse/whoinhouseassays.pdf.

All these kits are approved by the Indian Council of Medical Research for PCR testing purposes and the ranges of sensitivity >95% and specificity >99%. Also mentioned here LOD for each kit which varies.

Table S4 - PCR confirmed COVID cases and deaths of Mumbai COVID dashboard at different viral outbreak periods from April 2020 - January 2022

	Cases					Deaths				
			Outbreak periods					Outbreak periods		
Characteristics	Total since Apr 2020 to Jan 2022	Periods no major outbreaks	Aleph Wave: Jun-Nov 2020 (6 mo.)	Delta wave: Mar-Jun 2021 (4 mo.)	Omicron: Jan 2022 (till Jan17) (1 mo.)	Total since Apr 2020 to Jan 2022	No major outbreaks	Aleph wave: May-Oct 2020 (6 mo.)	Delta wave: Mar-Jun 2021 (4 mo.)	Omicron wave: Jan 2022 (till Jan17) (1 mo.)
No. of months	22	11	6	4	1	22	11	6	4	1
**PCR confirmed cases**										
**Total**	**1,022,979**	**160,288**	**232,484**	**376,528**	**253,679**	**16,668**	**2,850**	**9,627**	**3,980**	**211**
**Annualized rate per 1000 population****										
**All ages**	**192**	**13**	**34**	**82**	**222**	**1.5**	**0.2**	**1.4**	**0.9**	**0.2**
0-9	29	2	4	11	36	0.0	0.0	0.0	0.0	0.0
10-19	66	4	8	26	83	0.0	0.0	0.0	0.0	0.0
20-29	163	9	21	66	201	0.1	0.0	0.1	0.0	0.0
30-39	237	14	36	102	284	0.3	0.1	0.2	0.2	0.0
40-49	248	17	47	115	276	1.0	0.2	0.9	0.7	0.1
50-59	332	25	77	149	357	3.3	0.6	3.4	2.0	0.1
60-69	414	33	95	179	452	7.8	1.2	7.9	4.3	0.8
70-79	559	41	115	229	638	15.9	2.1	15.1	9.2	2.6
80+	588	39	104	247	688	23.3	3.2	19.5	14.0	6.1
**Sex**										
Female	n.a.	n.a.	29	76	192	n.a.	n.a.	n.a.	n.a.	n.a.
Male	n.a.	n.a.	38	88	248	n.a.	n.a.	n.a.	n.a.	n.a.
**Municipal ward, Slum density**										
**Low slum areas**	**261.2**	**16.4**	**45.1**	**123.5**	**262.5**					
A	379	23	55	132	484					
B	79	9	23	34	79	Deaths by wards not available				
C	101	8	37	46	93					
D	316	22	61	148	349					
E	155	15	33	73	162					
G South	193	16	38	81	219					
H West	470	25	53	159	625					
K West	324	17	38	116	422					
R Central	257	16	61	131	264					
T	266	22	66	138	261					
**Medium slum areas**	**173.8**	**13.1**	**32.8**	**77.4**	**195.7**					
F North	179	15	27	80	205					
F South	169	14	43	65	186					
G North	137	12	32	60	148					
M West	216	14	30	85	267					
N	149	12	34	74	152					
P South	270	14	39	123	319					
R South	209	12	41	100	231					
S	150	10	29	62	174					
**High slum areas**	**151.5**	**8.2**	**25.9**	**68.8**	**164.3**					
H East #	166	12	22	68	203					
K East	206	12	31	88	247					
L	99	8	16	40	119					
M East #	91	7	15	38	108					
P North	144	8	30	70	157					
R North	159	8	34	77	172					

Notes:

1. ** The denominator for case rate and death rates was the estimated 13 million Mumbai population. For convenience of comparing rates between viral waves, rates were adjusted to duration (number of months) of each pandemic wave to reflect the annual rate.
2. Data presented in this table were from Mumbai COVID dashboard data and as available for 24 wards of the Greater Mumbai municipal corporation area (MCGM (2022a)). COVID deaths by wards were not available.
3. # Ward H East and M East are home to Dharavi slums, one of the highest population density areas of Mumbai.
4. Wards with less than 33% of population lives in slums were categories as low slum density areas and more than 60% as high slum density areas. Detailed slum population shows in S1 Table.
5. Rates by sex was calculated using a subset of data where male and female breakdown was available.

Table S5 - Descriptive PCR positivity rates (%) of the Thyrocare tested population at different viral outbreak periods from April 2020 to January 2022

Characteristics	Total period	Aleph wave Jun-Nov 2020 (6 months)		Delta wave Mar-Jun 2021 (4 months)		Omicron wave Jan 2022 (1 months)		Periods no major outbreaks Apr 2020 - Jan 2022	
	No. tested (000)	No. tested (000)	***PCR Positivity % ****	No. tested (000)	***PCR Positivity % ****	No. tested (000)	***PCR Positivity % ****	No. tested (000)	***PCR Positivity % ****
Total no. tested (No. positive)	2,717.3 (155.0)	64.2 (17.9)	***23.2***	445.1 (46.6)	***9.9***	26.2 (11.7)	***42.8***	2,181.7 (78.8)	***3.4***
**Age in 10 years**									
0-9	73.7	0.9	***28.9***	14.1	***9.8***	0.7	***38.5***	58.0	***5.3***
10-19	269.5	2.3	***25.0***	39.6	***9.3***	1.5	***40.5***	226.1	***4.1***
20-29	641.5	13.4	***15.6***	103.2	***8.7***	5.9	***40.2***	518.9	***2.7***
30-39	580.5	15.7	***20.1***	99.2	***10.5***	7.0	***42.8***	458.6	***3.6***
40-49	457.7	11.1	***28.4***	75.6	***10.6***	4.0	***45.5***	367.0	***3.6***
50-59	329.2	9.2	***39.5***	53.7	***11.3***	3.1	***48.7***	263.2	***3.7***
60-69	233.0	6.5	***42.9***	37.1	***12.2***	2.2	***51.7***	187.2	***4.2***
70-79	106.7	3.7	***43.5***	17.9	***14.0***	1.2	***55.7***	84.0	***4.4***
80+	25.3	1.4	***41.2***	4.7	***20.5***	0.5	***55.9***	18.7	***6.1***
**Sex**									
Female	1,105.3	22.5	***25.9***	166.4	***11.5***	12.1	***44.1***	904.2	***3.8***
Male	1,612.0	41.7	***21.9***	278.7	***9.0***	14.1	***41.8***	1,277.5	***3.2***
***District, Municipal wards and slum* density**									
Mumbai	2,567.0	33.7	***22.0***	386.7	***7.8***	12.6	***41.3***	2,134.0	***3.4***
Mumbai Suburban	150.3	30.5	***24.7***	58.5	***23.5***	13.6	***44.2***	47.8	***6.5***
**Low slum areas**	**2,519.1**	**35.8**	**20.4**	**366.2**	**7.2**	**4.9**	**44.3**	**2,112.1**	**3.3**
A	2,386.8	6.1	7.8	321.9	5.6	0.3	35.2	2,058.5	3.3
B	12.6	4.1	46.6	5.5	19.7	0.0	43.5	2.9	5.2
C	2.7	0.6	11.4	0.8	15.5	0.2	35.0	1.0	4.9
D	48.2	6.0	16.0	14.7	12.8	0.8	42.1	27.5	4.2
E	11.8	0.9	9.4	5.8	17.3	-	-	4.3	3.3
GS	9.1	3.1	15.0	2.3	18.9	0.4	45.1	3.3	7.1
H West	6.1	1.2	18.6	2.5	20.6	0.3	47.5	2.1	6.8
K West	24.8	8.6	23.1	6.9	23.7	1.5	42.7	7.8	8.8
R Central	9.3	3.1	33.6	3.0	24.5	0.3	50.9	2.9	11.6
T	7.8	2.0	15.9	2.9	35.2	1.2	48.8	1.7	8.9
**Medium slum areas**	**79.1**	**11.8**	**28.8**	**30.9**	**25.3**	**9.2**	**42.2**	**27.3**	**8.3**
F North	11.8	3.2	38.4	4.4	24.6	0.9	47.0	3.3	21.7
F South	6.0	0.5	25.9	2.4	16.2	0.3	32.6	2.8	2.2
G North	21.2	3.7	26.3	7.0	20.5	2.7	37.0	7.9	7.3
M West	5.8	0.8	24.8	2.5	24.2	0.5	45.9	1.8	4.4
N	10.5	0.6	38.2	4.2	23.5	1.8	43.8	4.0	7.4
P South	10.0	1.6	15.1	4.4	30.5	1.3	38.6	2.7	8.0
R South	10.3	1.1	32.4	5.0	35.9	1.3	55.0	2.9	8.6
S	3.6	0.3	29.5	1.0	21.2	0.5	32.6	1.7	3.9
**High slum areas**	**119.0**	**16.6**	**25.8**	**48.0**	**19.9**	**12.1**	**42.8**	**42.3**	**5.2**
H East	36.0	5.9	21.6	16.2	16.4	2.4	36.2	11.5	3.7
K East	26.1	2.8	12.4	10.0	20.9	2.9	43.3	10.4	4.7
L	24.0	1.5	20.4	9.1	15.7	2.7	41.0	10.7	5.2
M East	8.2	1.7	23.8	2.8	19.4	0.9	43.9	2.8	8.1
P North	17.9	3.3	50.8	7.2	29.3	2.3	47.0	5.1	8.3
R North	6.8	1.4	34.9	2.7	29.2	0.8	56.0	1.9	6.5

Notes:

1. This table presents PCR test positivity of 2.7 million self-tested population in Mumbai. The last column "Periods no major outbreaks" are the remaining 10 months without major outbreaks.
2. * To compare PCR positivity rates between viral waves, we standardized the age of tested population to age distribution of census population in 2011.

Table S6 - Descriptive Ct values of the Thyrocare tested population during different viral waves from April 2020 to January 2022

Characteristics	Total period	Aleph wave Jun-Nov 2020 (6 mo.)		Delta wave Mar-Jun 2021 (4 mo.)		Omicron wave Jan 2022 (1 mo.)		Periods no major outbreaks Apr 2020 - Jan 2022	
	No. tested (000)	No. tested (000)	Median Ct value	No. tested (000)	Median Ct value	No. tested (000)	Median Ct value	No. tested (000)	Median Ct value
**Total**	**2,717.3**	**64.2**	**26.0**	**445.1**	**23.0**	**23.2**	**23.6**	**2,181.7**	**25.0**
**Age in 10 years**									
0-9	73.7	0.9	24.0	14.1	24.0	0.7	22.9	58.0	25.0
10-19	269.5	2.3	25.0	39.6	23.0	1.5	23.1	226.1	25.0
20-29	641.5	13.4	25.0	103.2	23.0	5.9	23.7	518.9	25.0
30-39	580.5	15.7	26.0	99.2	23.0	7.0	23.2	458.6	24.0
40-49	457.7	11.1	26.0	75.6	23.0	4.0	23.0	367.0	25.0
50-59	329.2	9.2	26.0	53.7	22.0	3.1	22.8	263.2	25.0
60-69	233.0	6.5	26.0	37.1	22.0	2.2	21.9	187.2	24.0
70-79	106.7	3.7	26.0	17.9	22.0	1.2	21.8	84.0	24.0
80+	25.3	1.4	26.0	4.7	22.0	0.5	21.4	18.7	24.0
**Sex**									-
Female	1,105.3	22.5	25.0	166.4	23.0	12.1	23.1	904.2	25.0
Male	1,612.0	41.7	26.0	278.7	23.0	14.1	23.2	1,277.5	24.0
**District, municipal wards and slum density**									
Mumbai	2,567.0	33.7	26.0	386.7	23.0	12.6	23.2	2,134.0	25.0
Mumbai Suburban	150.3	30.5	25.0	58.5	22.0	13.6	23.2	47.8	23.5
	**2,519.1**	**35.8**	**26.0**	**366.2**	**24.0**	**4.9**	**22.9**	**2,112.1**	**25.0**
**Low slum areas**									
A	2,386.8	6.1	29.0	321.9	24.0	0.3	23.6	2,058.5	25.0
B	12.6	4.1	26.0	5.5	22.0	0.0	20.8	2.9	26.0
C	2.7	0.6	26.0	0.8	23.0	0.2	25.5	1.0	22.1
D	48.2	6.0	28.0	14.7	24.0	-	-	27.5	26.0
E	11.8	0.9	27.0	5.8	23.0	0.8	23.3	4.3	22.8
G South	9.1	3.1	27.0	2.3	22.0	0.4	22.6	3.3	22.2
H West	6.1	1.2	25.0	2.5	22.0	0.3	23.5	2.1	22.0
K West	24.8	8.6	25.0	6.9	22.0	1.5	22.9	7.8	23.0
R Central	9.3	3.1	24.0	3.0	22.0	0.3	22.1	2.9	22.0
T	7.8	2.0	26.0	2.9	21.0	1.2	22.7	1.7	22.0
**Medium slum areas**	**79.1**	**11.8**	**25.0**	**30.9**	**21.0**	**9.2**	**23.0**	**27.3**	**23.0**
F North	11.8	3.2	26.0	4.4	21.0	0.9	23.1	3.3	22.0
F South	6.0	0.5	27.0	2.4	22.0	0.3	24.4	2.8	23.0
G North	21.2	3.7	25.0	7.0	21.0	2.7	23.3	7.9	23.0
M West	5.8	0.8	28.0	2.5	24.0	0.5	23.0	1.8	25.0
N	10.5	0.6	23.0	4.2	21.0	1.8	22.8	4.0	22.0
P South	10.0	1.6	25.0	4.4	21.0	1.3	22.7	2.7	22.0
R South	10.3	1.1	25.0	5.0	20.0	1.3	22.6	2.9	23.0
S	3.6	0.3	26.0	1.0	22.0	0.5	23.4	1.7	22.3
**High slum areas**	**119.0**	**16.6**	**25.5**	26.0	**22.5**	22.0	**23.7**	23.4	
H East	36.0	5.9	26.0	16.2	23.0	2.4	25.0	11.5	24.0
K East	26.1	2.8	25.0	10.0	22.0	2.9	23.1	10.4	22.0
L	24.0	1.5	26.0	9.1	22.0	2.7	23.2	10.7	23.0
M East	8.2	1.7	26.0	2.8	22.0	0.9	23.3	2.8	21.0
P North	17.9	3.3	25.0	7.2	22.0	2.3	22.8	5.1	24.0
R North	6.8	1.4	26.0	2.7	21.0	0.8	22.9	1.9	25.0

Notes:
This table presents PCR Ct values of 2.7 million self-tested population data from Thyrocare laboratory network. Total study period is 22 months from April 2020 to January 2022 and during this period 3 pandemic outbreaks were experienced in Greater Mumbai area. The last column "Periods no major outbreaks" is the remaining 11 months of the pandemic period where no major outbreaks occurred.

Table S7 - The begin, peak and end of viral waves observed in Ct values, PCR positivity, confirmed COVID case and deaths at Delta and Omicron outbreaks

Pandemic period/ Time series	The earliest date of change observed at			No. of days from the earliest observed date ¥			Number of days beginning to peak and peak to end		
	Beginning	Peak	End	Beginning	Peak	End	Beginning to peak	Peak to end	Overall
**Delta wave (Mar-Jun 2021)**									
**Thyrocare study data**									
Ct value	1-Feb-2021	31-Mar-2021	19-May-2021	0	0	0	58	49	107
PCR Positivity	18-Feb-2021	5-Apr-2021	16-Jun-2021	17	5	28	46	72	118
**MCGM official PCR confirmed**									
Cases	9-Feb-2021	5-Apr-2021	4-Jul-2021	8	5	46	55	90	145
Deaths	13-Mar-2021	1-May-2021	8-Aug-2021	40	31	81	49	99	148
**Omicron wave (Jan 2022)**									
**Thyrocare study data**									
Ct value	11-Dec-2021	2-Jan-2022	30-Jan-2022*	0	0	*	22	28*	50*
PCR Positivity	22-Dec-2021	11-Jan-2022	30-Jan-2022*	11	9	*	20	19*	39*
**MCGM official PCR confirmed**									
Cases	18-Dec-2021	10-Jan-2022	29-Jan-2022*	7	8	*	23	19*	42*
Deaths	4-Jan-2022	24-Jan-2022	30-Jan-2022*	24	22	*	20	6*	26*

Notes:
This table presents wave properties of the earliest dates observed in the beginning, peak and end of Ct values, PCR positivity, MCGM's confirmed COVID case prevalence and death time series during Delta and Omicron SARS-CoV-2 outbreak periods in Mumbai. Each time series was recorded daily and used 7-day average for this analysis. The relevant daily time series data are illustrated in S2 Figure. Aleph wave (Jun - Nov 2020) was excluded here because Ct values were not available for the complete outbreak period.
* In Omicron wave, end date would not be fully accurate because data collection terminated prematurely at end of January 2022.
¥ No. of days were calculated using the difference of days between the earliest observed date of any of the time series.

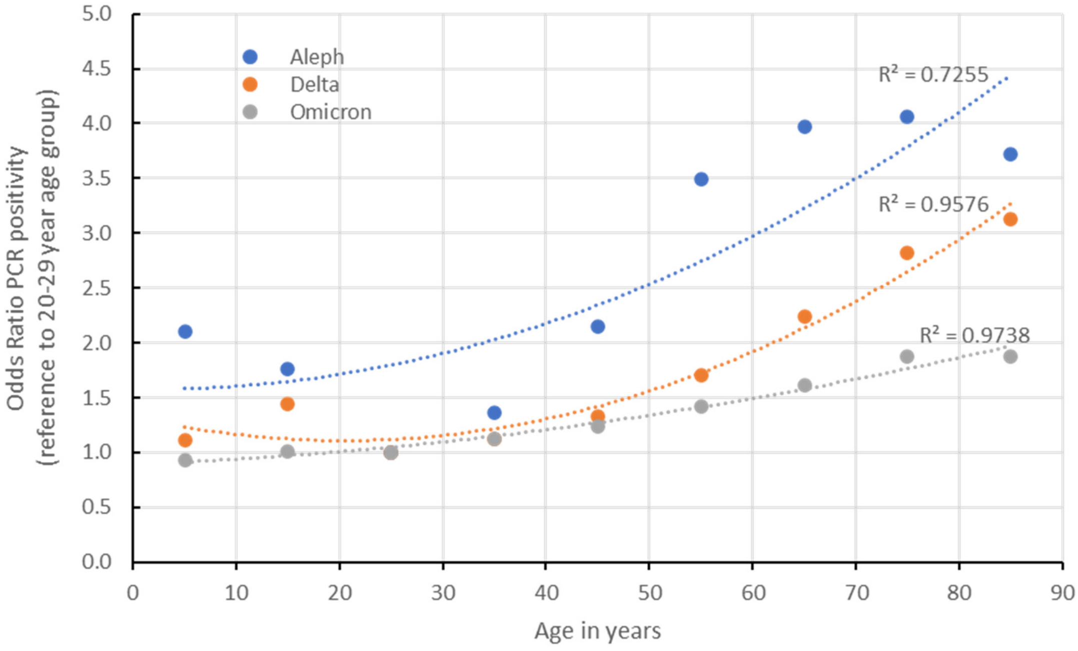

Figure S1 - Age trend of PCR positivity during Aleph, Delta, and Omicron viral waves

The dotted scatter represents the gender and slum status adjusted odds ratios of PCR positivity for 10 yearly age groups. Odds ratios were measured relative to the age 20-29 year group whom always had the lowest positivity rate. The dotted curves are the polynomial trends for each viral wave. R-squared for each viral wave: Aleph 0.73, Delta 0.96 and Omicron 0.97. The original odds ratios used in this figure were extracted from Figure 3, forest plot. We tested for trend of age using Cochran-Armitage Trend Test: Aleph (p<0.0001), Delta (p<0.0001) and Omicron (p<0.0001).

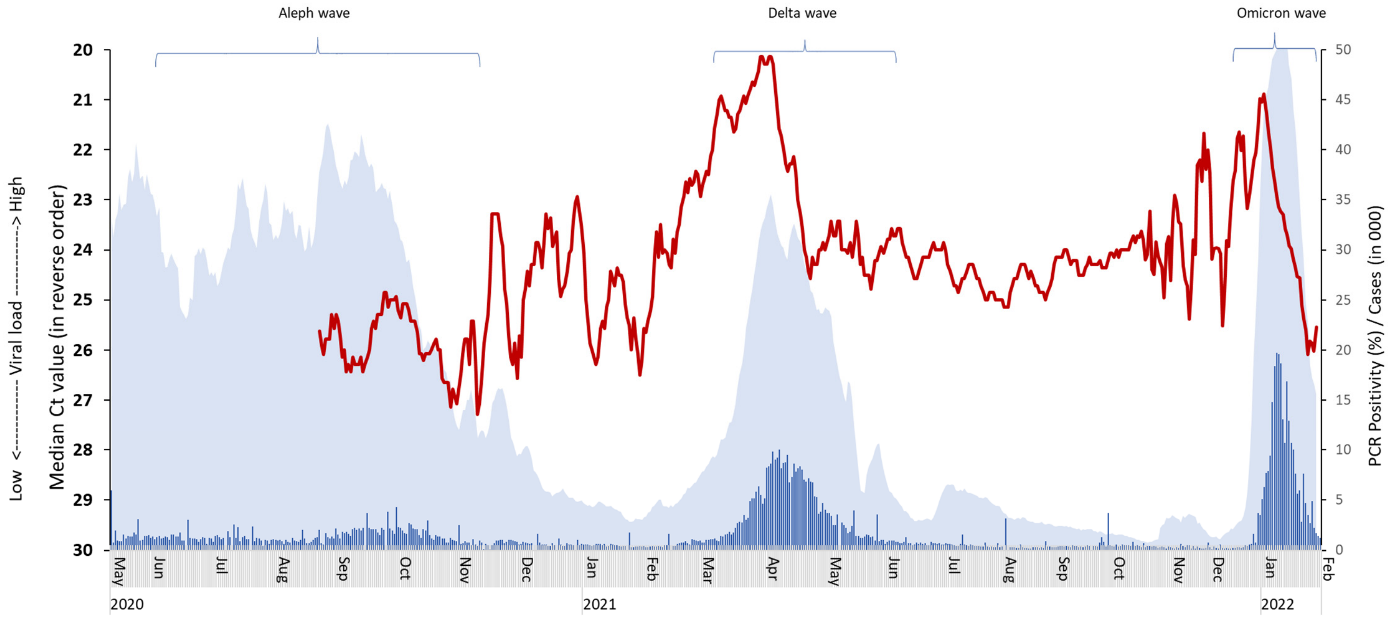

Figure S2 - Overview of Mumbai SARS-CoV-2 viral outbreaks - daily time series of MCGM COVID-19 dashboard and Thyrocare study data

First pandemic outbreak was from Jun-Nov 2020 (Aleph wave), second from Mar-Jun 2021 (Delta wave), and third in January 2022 (Omicron wave). Daily PCR confirmed case counts in 000 (blue bar) are as reported in Mumbai COVID dashboard. 7-day average PCR positivity (gray area) and daily median Ct values (red curve) are from Thyrocare data. The red curve represents the median Ct values refers to Y-axis on the left and all other time series refer to Y-axis on the right. Left Y-axis is shown in reverse order so a lower Ct value implies a higher viral load.

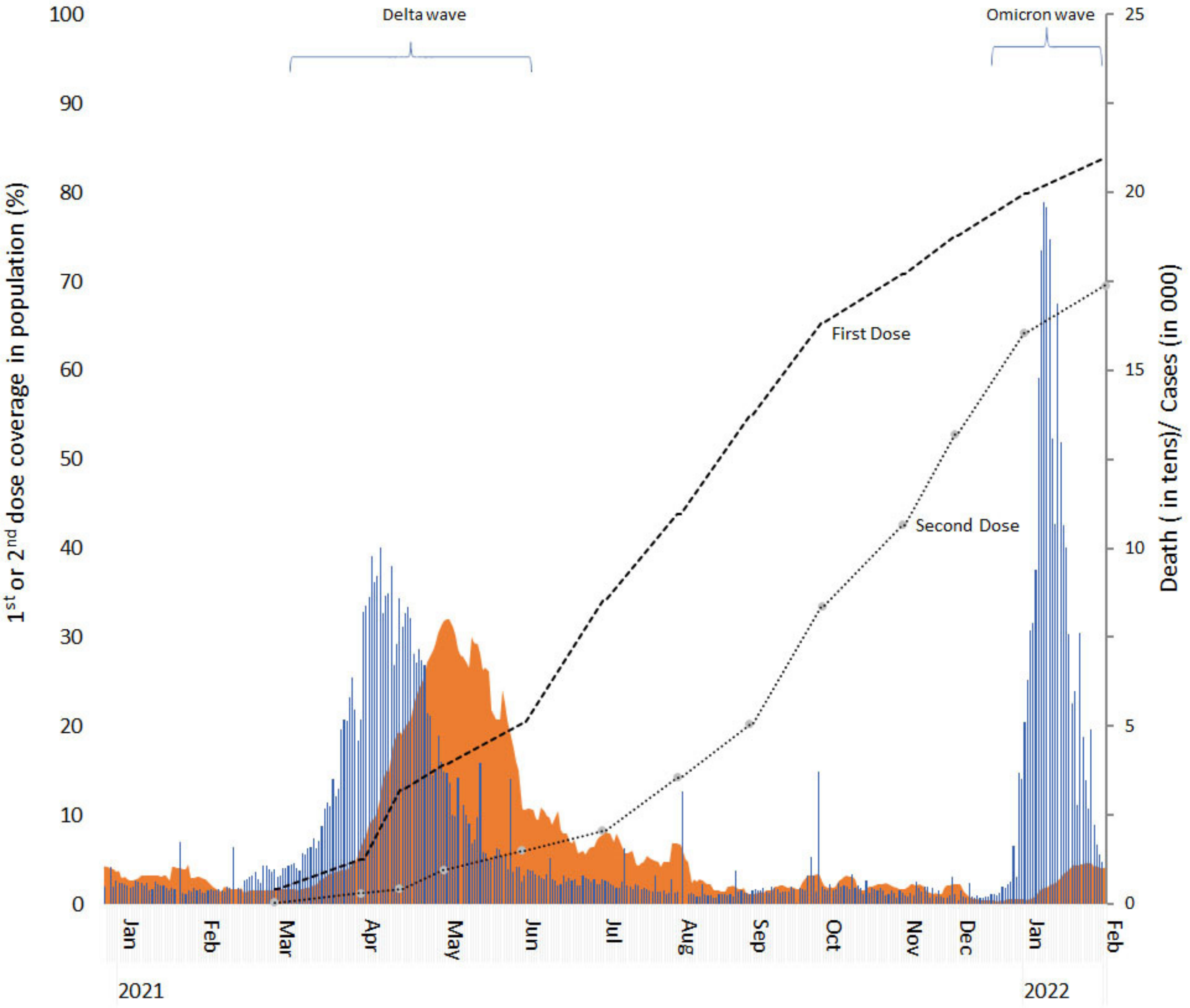

Figure S3 - Vaccine coverage, daily time series of PCR confirmed cases and deaths from COVID-19 from January 2021 - January 2022 in Mumbai

The first pandemic outbreak wave was from Jun-Nov 2020 (Aleph wave), second from Mar-Jun 2021 (Delta wave), and third in January 2022 (Omicron wave). First pandemic wave is not shown here because vaccine coverage was not substantial at the period. Daily PCR confirmed case counts in 000 (blue bar), deaths in tens (orange area), percentage of vaccine coverage (thick dotted line - 1st dose, thin dotted line - 2nd dose) are as reported in Mumbai COVID dashboard. The vaccine coverage refers to Y-axis in left and all other time series refer to Y-axis in right.
